# Comparative Analysis of HRTFs Measurement Using In-Ear Microphones

**DOI:** 10.3390/s23136016

**Published:** 2023-06-29

**Authors:** Valeria Bruschi, Alessandro Terenzi, Nefeli A. Dourou, Susanna Spinsante, Stefania Cecchi

**Affiliations:** Department of Information Engineering, Università Politecnica delle Marche, 60131 Ancona, Italy; v.bruschi@univpm.it (V.B.); a.terenzi@staff.univpm.it (A.T.); n.a.dourou@staff.univpm.it (N.A.D.); s.spinsante@staff.univpm.it (S.S.)

**Keywords:** head-related transfer function, head-related impulse response, HRTF measurement, individual HRTF measurement

## Abstract

The head-related transfer functions (HRTFs) describe the acoustic path transfer functions between sound sources in the free-field and the listener’s ear canal. They enable the evaluation of the sound perception of a human being and the creation of immersive virtual acoustic environments that can be reproduced over headphones or loudspeakers. HRTFs are strongly individual and they can be measured by in-ear microphones worn by real subjects. However, standardized HRTFs can also be measured using artificial head simulators which standardize the body dimensions. In this paper, a comparative analysis of HRTF measurement using in-ear microphones is presented. The results obtained with in-ear microphones are compared with the HRTFs measured with a standard head and torso simulator, investigating different positions of the microphones and of the sound source and employing two different types of microphones. Finally, the HRTFs of five real subjects are measured and compared with the ones measured by the microphones in the ear of a standard mannequin.

## 1. Introduction

The head-related transfer functions (HRTFs) are mathematical functions that represent the acoustic path between a sound source and the ears of a listener in the frequency domain. These functions can be expressed also in the time domain under the name of head-related impulse responses (HRIRs). Humans can perceive the direction and the distance of a sound source by evaluating the differences in sound between the ears. These differences are represented by the localization cues, such as interaural time difference (ITD), interaural level difference (ILD), and spectral cues, that are contained in the HRTFs [1]. For this reason, HRTFs are essential to understand the human being sound perception and they are used for binaural reproduction to enrich the acoustic signals with directional cues. Spatial audio systems can be obtained by binaural recordings or by processing the recorded signal with HRTFs [2]. In the second case, the binaural synthesis is achieved by the real-time convolution of the input signal with the respective HRIRs. Moreover, the increasing interest in deep learning has recently paved the way for machine learning (ML) methods for spatial audio processing [3]. ML algorithms can be applied for HRTF individualization to enhance the binaural rendering [4].

The HRTFs are strongly individual because they depends on the shape of head, pinnae, and torso that are different for each human being. However, mannequins with standardized dimensions can be used to generate standardized HRTFs [5]. Standard head and torso simulators are widely used in the literature to create HRTFs databases [6] and to investigate measurement limits, such as the directional resolution [7,8] and the distance between the sound source and the head [9,10]. However, there are individual differences in HRTFs that cannot be identified by the standardization. This problem can be solved by using in-ear microphones fitted to real subjects in order to measure individual (or personalized) HRTFs [11,12,13,14,15,16,17].

Several perceptual studies have aimed at evaluating how the use of individual HRTFs enables a high fidelity of the binaural rendering [18,19,20], resulting in contrasting conclusions. In fact, the analysis conducted in [18] shows that the personalized HRTFs are not always the preferred ones, while the studies presented in [19,20] prove that the source localization improves when individual HRTFs are involved. Although the use of individual HRTFs may represent the best solution in binaural reproduction, the HRTFs measurement could be affected by several errors. In [11], the effect of head movements during the HRTFs measurement has been evaluated, proving that the pitch movement, i.e., up or down movement of the head, is the largest among roll, pitch, and yaw, and could cause spectral differences of the HRTFs up to 6 dB. To manage this drawback, a neck support could be applied to the listener under measurement [12], or databases of individual HRTFs may be created including information about the azimuth and the elevation of the sound source and the orientation of the listener’s head, captured by a tracking system [13,14].

Another important aspect of individual HRTFs measurements is the microphone position [15,21]. Actually, the sound pressure distribution along the ear canal is non-uniform [22,23,24,25,26], so different microphone positions produce different measurement results that are not directly comparable. Depending on the type of study, the microphone could be placed at the entrance of the ear canal [5,27,28], inside the ear canal [22,29] or close to the eardrum [23,30,31]. Several researches have proven the necessity to record the signal near the eardrum, because in this way the HRTF measurements include most of the localization cues and the ear canal resonance [32,33,34]. However, positioning miniature microphones in a fixed and replicable position inside the ear canal could be very difficult. In [32], the ear canal is modeled as a one-dimensional transmission line, considering it as the direction-dependent part. Moreover, the psychoacoustic analyses carried out in [33] have proven that the sound direction depends only on the acoustic path between the sound source and the ear canal entrance. In [33], the acoustic transfer path from the sound source and the eardrum is divided into three parts, one direction-dependent part (from the source to the ear canal entrance), two direction-independent parts (from the blocked to open entrance of the ear canal), and the transmission along the ear canal.

In this context, this paper presents a new comparative analysis of two different in-ear miniature microphone systems positioned inside the ear canal for the HRTF measurement. Taking into account different positions of a sound source (i.e., varying the azimuth and the elevation), two analyses have been carried out. A comparison between the HRTFs obtained with the proposed systems and the HRTFs measured by a standard binaural mannequin is performed first, then an analysis of several HRTFs measured considering real subjects is reported. The former is performed to study the performance of the acquisition systems and the effect of the microphones positions with respect to the ear canal. The latter is performed to evaluate the performance of these two different systems considering real subject ear’s shape. All these analyses have been performed in terms of objective evaluations.

The paper is organized as follows. Section 2 offers an overview of the most used measurement algorithms for the impulse response acquisition. Section 3 widely explains the hardware used for the experiments. Section 4 shows the experimental results and a detailed analysis of the measurements. Finally, Section 5 reports the conclusions.

## 2. Measurement Techniques

The head-related impulse responses (HRIRs) can be measured using several approaches that can be divided into two groups, deconvolution methods and adaptive filtering techniques [21]. The deconvolution methods are the most popular and can be, in turn, classified depending on the input signals, as pseudo random sequences [17,35,36,37,38,39,40,41,42,43] or sweep signals [44,45,46,47,48]. The pseudo random sequences include the maximum length sequence (MLS) [36,37,38,39,40], the inverse repeated sequence (IRS) [41,42] and the Golay codes [43]. In [49], a comparison of the most used deconvolution methods can be found, and in [50], the MLS method is compared with the sweep. The other measurement approach is based on the adaptive filtering technique, employed in HRTFs measurements for the first time in [51] and then applied also in [10,52]. In most of the cases, the adaptation procedure is performed using the normalized least mean square (NLMS) algorithm, thanks to its simplicity and high performance [53].

HRTF measurements could be affected by several problems, such as non-linear distortions of the electro-acoustic systems, environmental noises, reflections from the environments, sound source characteristics, and temperature variations [21,54]. The measurement inside a controlled environment (e.g., anechoic chamber) can solve the problems derived by the environment, while non-linear distortions can be avoided by choosing the appropriate procedure and stimuli [55,56,57]. In [58], perfect periodic sequences (PPSs) and orthogonal periodic sequences (OPSs) are applied for HRTFs measurement in a real car environment, proving robustness towards non-linearities. In particular, PPSs are periodic sequences that require the perfect orthogonality of the basis functions over a period, so they can be used for the identification of Legendre non-linear (LN) filters [56,59] or Wiener non-linear (WN) filters [60,61]. Similarly, the OPSs is a periodic sequence that can identify functional link polynomial (FLiP) filters, i.e., a wide class of non-linear filters that includes LN and WN filters [62,63,64].

For the comparative analysis proposed in this paper, the measurements have been carried out in a semi-anechoic environment with professional equipment and low levels of input signals to reduce possible distortions introduced by the system. The impulse responses have been measured using the sweep signal. Further details on the hardware setup and the acquisition chain are reported in the next section.

## 3. Hardware Setup

**Figure 1:**
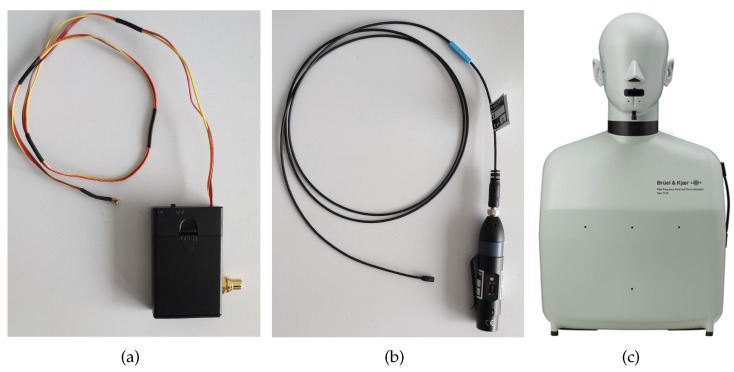
(**a**) Knowles FG-23329-D65 with its battery power supply, (**b**) Sennheiser MKE2-EW Gold microphone with its power supply and signal conditioner MZA900P, and (**c**) Brüel & Kjær head and torso simulator (HATS) Type 4128C.

**Figure 2:**
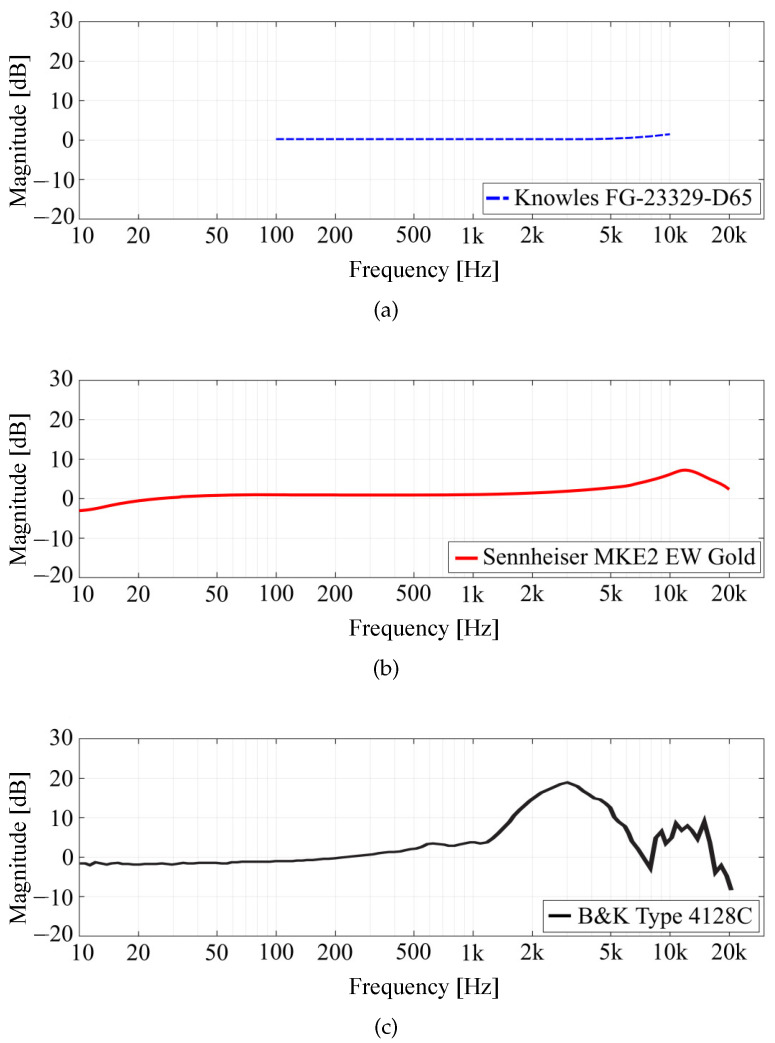
Frequency responses of (**a**) Knowles FG-23329-D65 [65], (**b**) Sennheiser MKE2 EW Gold [66], and (**c**) Brüel & Kjær head and torso simulator (HATS) Type 4128C [67], provided by the manufacturers.

Focusing on in-ear microphones, the sensors used must be as small as possible, indeed this is important for two main reasons; first, since the sensor is placed inside the ear canal, a small device could be installed without being too much annoying for the subject, then, a small form factor is also important in order to minimize any modification in the ear form which can degrade the quality of the measured responses. Another important aspect of the microphone is its frequency response, which should be as flat as possible at least until 10 kHz. For this analysis, the HRTF measurements have been carried out with two different microphones, i.e., the Knowles FG-23329-D65 and the Sennheiser MKE2-EW Gold. Figure 1a,b shows photos of the microphones and Figure 2a,b show their frequency responses. The Knowles FG-23329-D65 [65] is an electret condenser omnidirectional microphone. Its dimensions are about a few millimeters in diameter, as shown in Figure 1a, enabling an easy placement inside the ear canal. The Knowles microphone has a very flat frequency response in a reasonable band between 100 Hz and 10 kHz, as visible in Figure 2a. It has also a very limited power consumption (50 μA), so just two AA batteries are needed to power the microphone, avoiding noise problems. The Sennheiser MKE2-EW [66] Gold is a condenser Lavalier omnidirectional microphone. It features a wide frequency range, from 20 Hz to 20 kHz, as reported in Figure 2b, and an almost flat frequency response below 5 kHz. Figure 1b shows the microphone with its power supply/signal conditioner Sennheiser MZA-900P. The microphone is powered by a 48 V phantom line and generates a low-impedance balanced output. In comparison with the Knowles, the Sennheiser features a slightly bigger capsule with a thicker wire which makes the placement more difficult, on the other side the Sennheiser has a wider frequency response and more robust construction and it can be easily powered by any modern soundcard. On the other hand, the Knowles microphone price is one order of magnitude lower than Sennheiser microphone.

**Figure 3:**
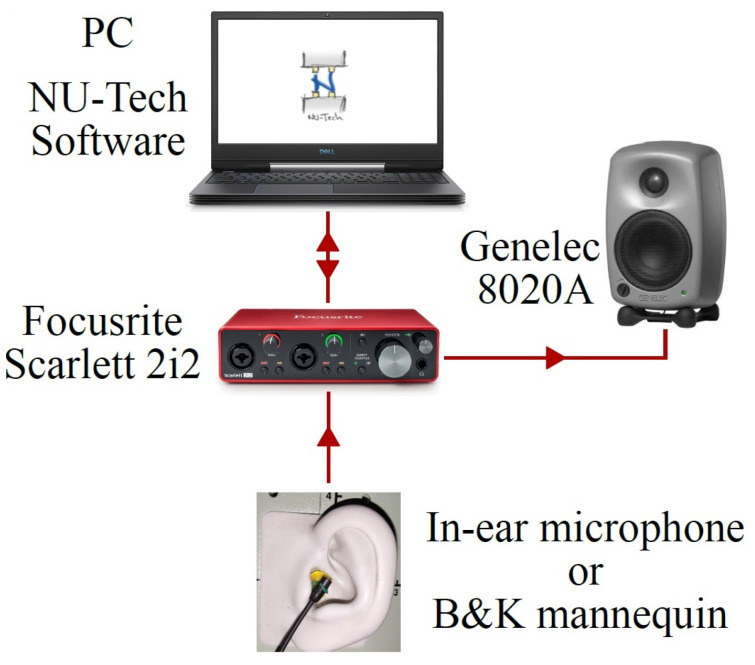
Scheme of the acquisition chain used for HRTF measurements.

**Figure 4:**
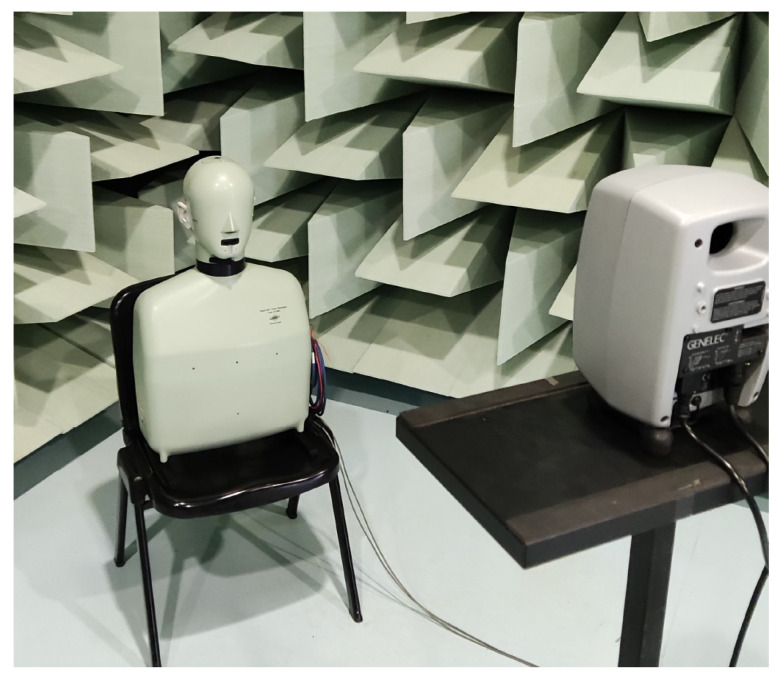
Photo of the experimental setup used for HRTF measurements.

The HRTFs measured with the two microphones are compared with the ones measured with the Brüel & Kjær head and torso simulator (HATS) Type 4128C, i.e., a binaural mannequin used as a reference and shown in Figure 1c. The frequency response of the mannequin is reported in Figure 2c. In this case, the magnitude response is not flat due to the effect of the ear of the dummy head. For the measurements with the B&K simulator, the mannequin is connected to its power supply B&K PS 2829. Moreover, the microphones and a Genelec 8020 A are connected to the Scarlett Focusrite 2i2 soundcard, managed by a computer that uses the NU-Tech software (version 2.0) [68] for the acquisitions. The frequency response of the Genelec loudspeaker is declared flat (±2.5 dB) in the frequency range of 66 Hz–20 kHz by the manufacturer. The measurements have been carried out inside a semi-anechoic chamber and taking into account only the left ear. The scheme of the acquisition chain used for HRTF measurements is shown in Figure 3. A photo of the experimental setup is shown in Figure 4.

The non-linearities that the measurement system may introduce have been evaluated by calculating the total harmonic distortion (THD) on a tone at 1 kHz. The THD is defined as the percentage ratio between the power of all harmonics and the power of the fundamental frequency. The THD values obtained with the three microphones are reported in the following:

- For the Brüel & Kjær HATS simulator, THD=0.9%;
- For the Knowles microphone, THD=1.8%;
- For the Sennheiser microphone, THD=0.4%.

The highest distortion is obtained with the Knowles microphone and it is lower than 2%, so the system can be assumed linear. In addition, the impulse response measurements have been performed using a logarithmic sweep signal thanks to its rejection of the harmonic distortions, as declared in [49]. The sweep used for the experiments has a length of 32,768 samples (i.e., 682.7 ms) and is repeated three times. The sampling frequency is $F_{s}=48$ kHz and the final impulse responses have a length of 4096 samples.

## 4. Experimental Results

**Figure 5:**
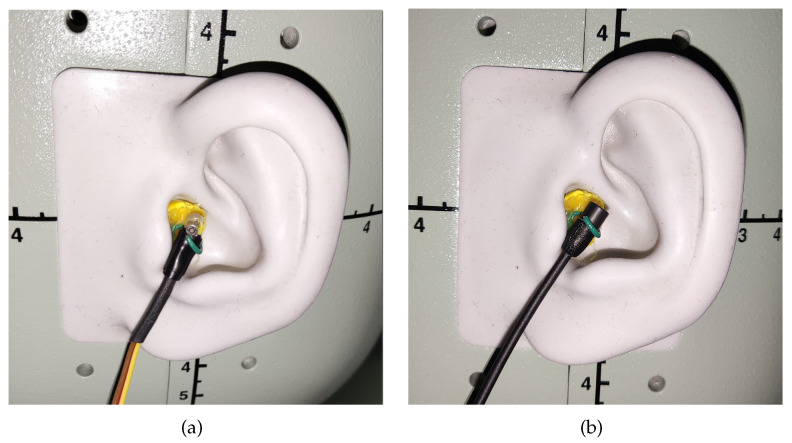
Photos of the (**a**) Knowles and (**b**) Sennheiser microphones on the mannequin ear.

**Figure 6:**
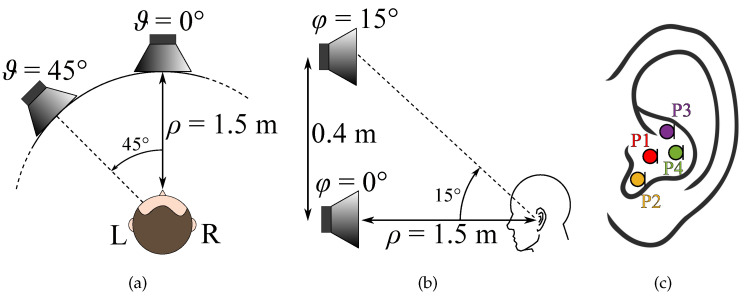
Position of the sound source in terms of (**a**) azimuth and (**b**,**c**) positions of the in-ear microphone considered in experiment 1. The microphone’s positions are identified with different colors (i.e., red for P1, yellow for P2, violet for P3, and green for P4).

**Figure 7:**
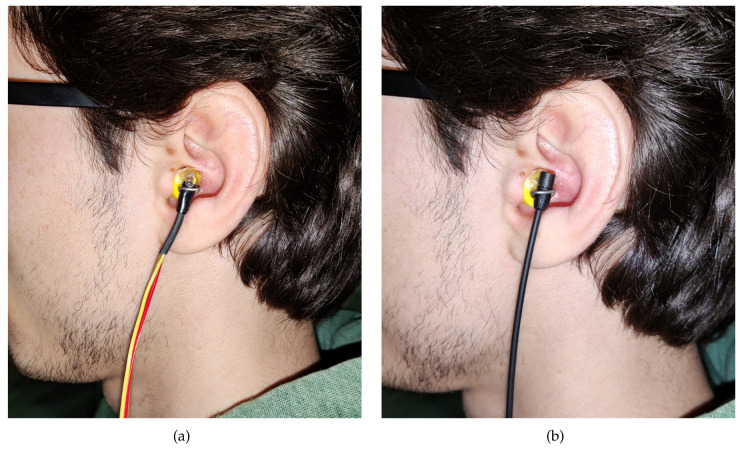
Photos of the (**a**) Knowles and (**b**) Sennheiser microphones on the ear of a real subject.

Two types of experiments have been carried out, in particular:

1. A comparison between the HRTFs measured with the in-ear miniature microphones placed in different points on the B&K mannequin ear canal and the HRTFs measured by the internal microphone of mannequin considering different positions of the sound source (see Figure 5 and Figure 6);
2. A comparison of individual HRTFs measured on five real subjects with the two in-ear microphones for different positions of the sound source (see Figure 7).

The two microphones have been settled on the left ear of the mannequin and of the subjects by means of a hook fixed on earplugs, as shown in Figure 5 and Figure 7. The measurements carried out with the in-ear microphones have been compared with the ones executed with the mannequin. It must be underlined that the comparison has been performed taking into consideration that the placement of the in-ear microphone occludes the ear canal, which is open when the HATS simulator is used.

For an objective evaluation, the HRTFs measured with the in-ear microphones are evaluated in terms of frequency magnitude response and log-spectral distance (LSD) [58] considering the B&K mannequin as reference. The LSD quantifies the distance between two spectra and, in this case, it is used to evaluate how much the HRTFs measured by the in-ear microphones differ from the ones measured with the mannequin. In particular, the LSD is calculated between the reference HRTF of the dummy ear $H_{\mathrm{HATS}}\left(k\right)$ and the one measured with the in-ear microphone $H_{\mathrm{MIC}}\left(k\right)$ as follows

(1) $$\mathrm{LSD}=\sqrt{\frac{1}{k_{2}-k_{1}+1}\sum_{k=k_{1}}^{k_{2}}{\left[10\log_{10}\frac{|H_{\mathrm{HATS}}\left(k\right){|}^{2}}{|H_{\mathrm{MIC}}\left(k\right){|}^{2}}\right]}^{2}},$$

where $k_{1}$ and $k_{2}$ delimit the frequency range within which the LSD is estimated, defined as $B=\left[k_{1}\frac{F_{s}}{K},\;k_{2}\frac{F_{s}}{K}\right]=\left[100\;\mathrm{Hz},\;10\;\mathrm{kHz}\right]$, with K=4096 the number of frequency bins for the FFT computation, and $F_{s}=48$ kHz the sampling frequency.

### 4.1. Experiment 1

The first experiment aims at analyzing the differences between the HRTFs measured with the mannequin and the HRTFs measured with the in-ear microphones settled on the dummy ear. The in-ear microphones have been placed in the mannequin ear considering four different positions, as shown in Figure 6c. Figure 5 shows the real microphone placement for position P1. Furthermore, four different positions of the sound source have been taken into account varying the azimuth ϑ and the elevation φ, as shown in Figure 6a,b, i.e., $\vartheta =0^{\circ },45^{\circ }$, and $\varphi =0^{\circ },15^{\circ }$.

**Figure 8:**
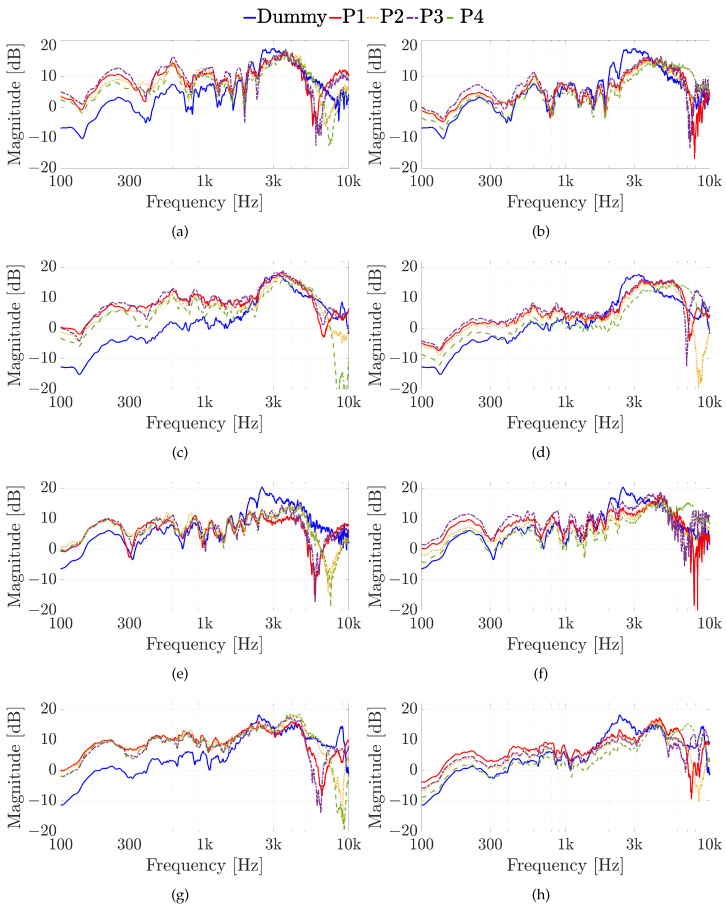
Experiment 1: HRTFs comparison considering the different in-ear microphone positions of Figure 6c for sound source positions (**a**,**b**) with $\vartheta =0^{\circ }$ and $\varphi =0^{\circ }$, (**c**,**d**) with $\vartheta =45^{\circ }$ and $\varphi =0^{\circ }$, (**e**,**f**) with $\vartheta =0^{\circ }$ and $\varphi =15^{\circ }$, and (**g**,**h**) with $\vartheta =45^{\circ }$ and $\varphi =15^{\circ }$, using (**a**,**c**,**e**,**g**) the Knowles microphone, and (**b**,**d**,**f**,**h**) the Sennheiser microphone.

**Figure 9:**
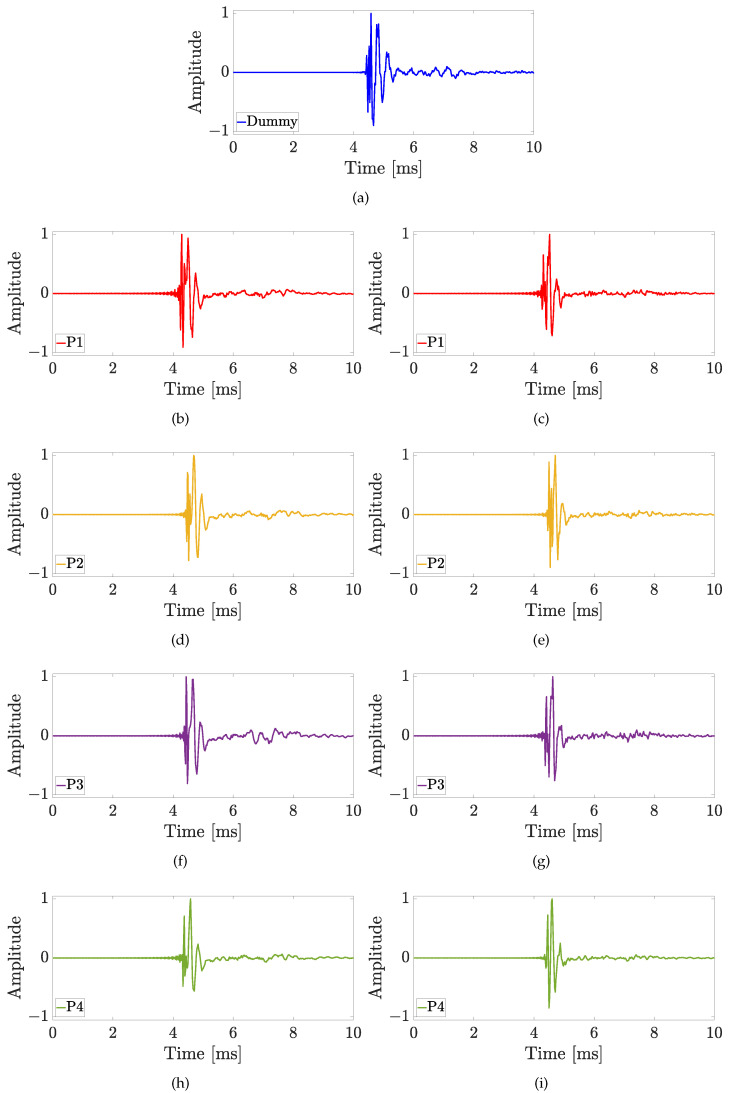
Experiment 1: Impulse responses comparison considering the different in-ear microphone positions of Figure 6c for the sound source position with $\vartheta =0^{\circ }$ and $\varphi =0^{\circ }$, using (**a**) the HATS, (**b**,**d**,**f**,**h**) the Knowles microphone, and (**c**,**e**,**g**,**i**) the Sennheiser microphone.

The aim of this experiment is to investigate how much the microphone position influences the HRTF measurement and the results are shown in Figure 8 for both the Knowles (in the first column) and the Sennheiser (in the second column) microphones, considering the four different positions of the sound source. The LSD values calculated for the first experiment are reported in Table 1. For the source position with $\vartheta =0^{\circ }$ and $\varphi =0^{\circ }$, the HRTFs measured by the Sennheiser microphone are more similar to the HRTF measured with the dummy head, especially at the low frequencies up to 2 kHz (cf. Figure 8a,b). In fact, the Sennheiser reaches the lowest values of the LSD for the first three positions of the microphone. Moreover, the microphone position with the lowest LSD is P2 for both Knowles and Sennheiser microphones, exhibiting values of 1.4 dB and 1.1 dB, respectively, (cf. Table 1). In this case, also the HRIRs in the time domain measured by the HATS and by the two microphones placed at different points are shown in Figure 9. The comparison among the time-domain impulse responses is more difficult than in the frequency domain and the differences are not so clear. However, in all the HRIRs, can be identified the direct pulse and the first reflection caused by the ear’s pinna, followed by the head and torso reflections up to 7 ms. Finally, the small late reflections above 7 ms are created by the measurement devices and the ground. Regarding the source position with $\vartheta =45^{\circ }$ and $\varphi =0^{\circ }$, the HRTFs measured with the Sennheiser are closer to the one measured with the mannequin at the low frequencies (cf. Figure 8c,d). However, the differences at higher frequencies produce higher LSD values with the Sennheiser, when the microphone is in positions P2 and P3. In this case, the best position is P3 for the Knowles with an LSD of 1.3 dB, and P1 for the Sennheiser with an LSD of 1.1 dB (cf. Table 1). For the source position with $\vartheta =0^{\circ }$ and $\varphi =15^{\circ }$, the Knowles microphone introduces a notch around 6 kHz for P1 and P3, and around 7.5 kHz for P2 and P4 (cf. Figure 8e,f), resulting in LSD values lower than the ones obtained with the Sennheiser microphone. The lowest LSD reached by the Knowles microphone is 1.5 dB in positions P2 and P3, while the lowest LSD for the Sennheiser is 0.9 dB in position P2 (cf. Table 1). Finally, for the source position with $\vartheta =45^{\circ }$ and $\varphi =15^{\circ }$, the HRTFs measured with the Sennheiser produce the best results both in terms of frequency response and LSD values (cf. Figure 8g,h). For the last source position, the lowest LSD with the Knowles microphone is 1.9 dB at point P1, and with the Sennheiser microphone is 1.3 dB at point P3 (cf. Table 1). These results prove that the position of the microphone affects the frequency response only at frequencies higher than 4 kHz and the performance of the Sennheiser microphone reaches the lowest LSD values in comparison with the Knowles microphone in most of the cases.

### 4.2. Experiment 2

**Figure 10:**
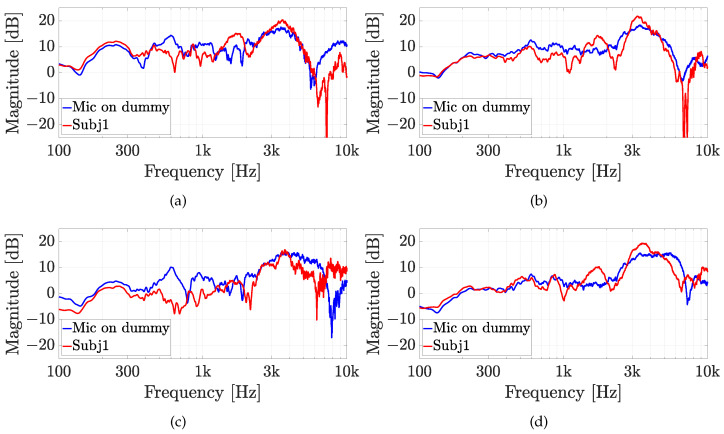
Experiment 2: HRTFs comparison of subject 1 with (**a**,**b**) the Knowles microphone and (**c**,**d**) the Sennheiser microphone for (**a**,**c**) $\vartheta =0^{\circ }$, and (**b**,**d**) $\vartheta =45^{\circ }$ and a fixed elevation of $\varphi =0^{\circ }$. The measurements are compared with the same microphone on the dummy ear.

**Figure 11:**
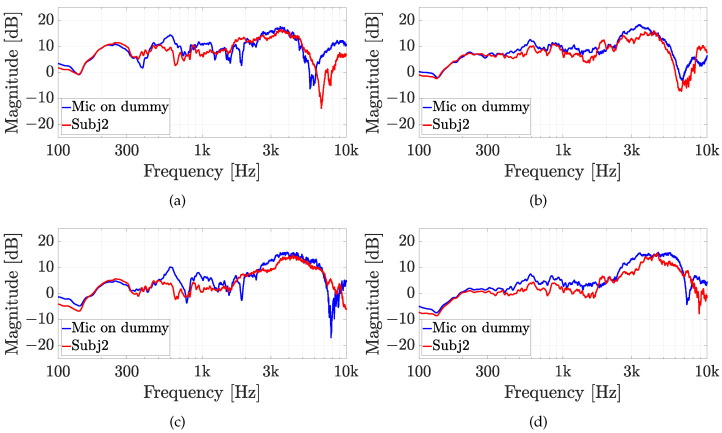
Experiment 2: HRTFs comparison of subject 2 with (**a**,**b**) the Knowles microphone and (**c**,**d**) the Sennheiser microphone for (**a**,**c**) $\vartheta =0^{\circ }$, and (**b**,**d**) $\vartheta =45^{\circ }$ and a fixed elevation of $\varphi =0^{\circ }$. The measurements are compared with the same microphone on the dummy ear.

**Figure 12:**
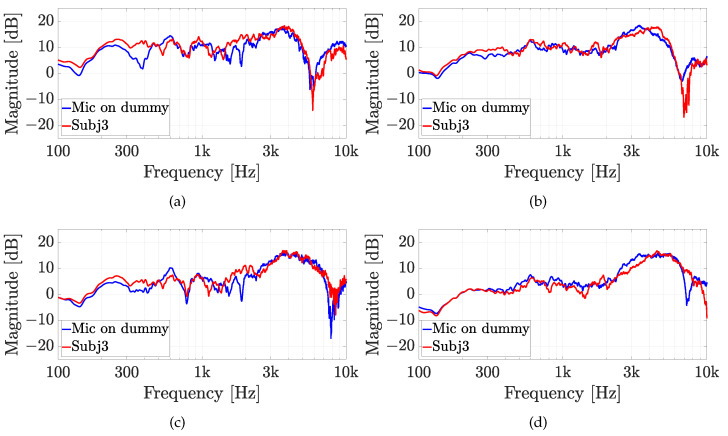
Experiment 2: HRTFs comparison of subject 3 with (**a**,**b**) the Knowles microphone and (**c**,**d**) the Sennheiser microphone for (**a**,**c**) $\vartheta =0^{\circ }$, and (**b**,**d**) $\vartheta =45^{\circ }$ and a fixed elevation of $\varphi =0^{\circ }$. The measurements are compared with the same microphone on the dummy ear.

**Figure 13:**
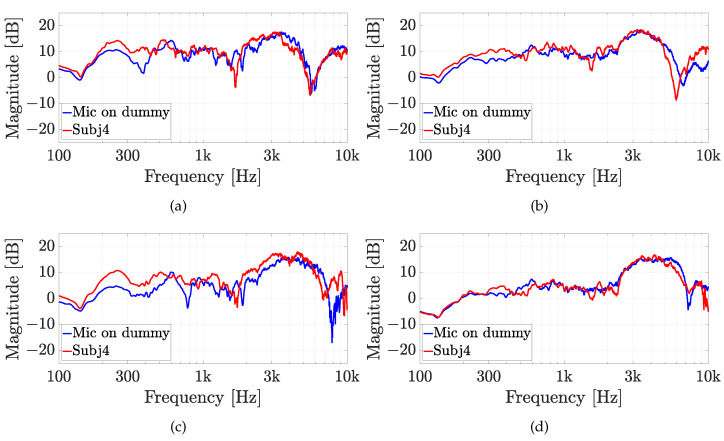
Experiment 2: HRTFs comparison of subject 4 with (**a**,**b**) the Knowles microphone and (**c**,**d**) the Sennheiser microphone for (**a**,**c**) $\vartheta =0^{\circ }$, and (**b**,**d**) $\vartheta =45^{\circ }$ and a fixed elevation of $\varphi =0^{\circ }$. The measurements are compared with the same microphone on the dummy ear.

**Figure 14:**
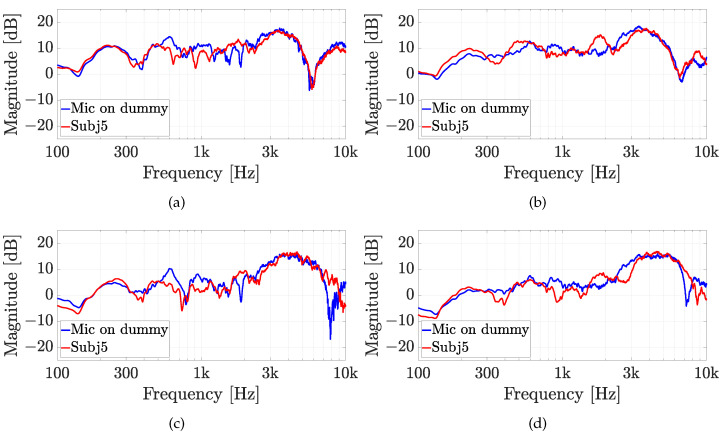
Experiment 2: HRTFs comparison of subject 5 with (**a**,**b**) the Knowles microphone and (**c**,**d**) the Sennheiser microphone for (**a**,**c**) $\vartheta =0^{\circ }$, and (**b**,**d**) $\vartheta =45^{\circ }$ and a fixed elevation of $\varphi =0^{\circ }$. The measurements are compared with the same microphone on the dummy ear.

**Figure 15:**
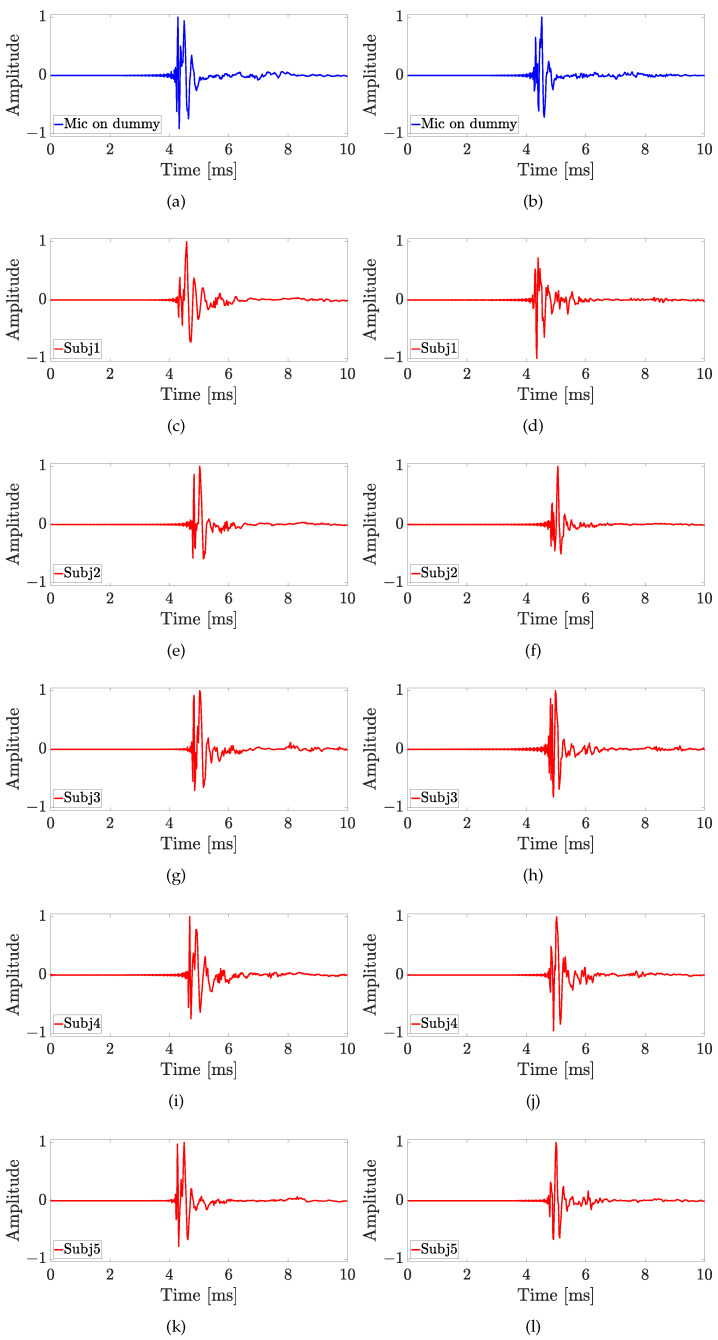
Experiment 2: Impulse responses measured with the Knowles microphone (first column) and the Sennheiser microphone (second column) on (**a**,**b**) the dummy head, and (**c**–**l**) on five real subjects for the sound source position with $\vartheta =0^{\circ }$ and $\varphi =0^{\circ }$.

The second experiment involves five real subjects wearing alternatively the two in-ear microphones, as shown in Figure 7. Results of experiment 2 are reported for each subject in Figure 10, Figure 11, Figure 12, Figure 13 and Figure 14, considering two azimuth angles of the sound source, i.e., $\vartheta =0^{\circ }$ and $\vartheta =45^{\circ }$ and an elevation of $\varphi =0^{\circ }$, as shown in Figure 6a. The measurements on subjects are compared with the HRTFs measured with the same microphone fixed on the dummy ear. In this case, the central position P1 of Figure 6c has been chosen for the acquisitions. As expected, each subject has a different frequency response due to the ear’s shape but a comparison between the two microphones and the dummy ear can be performed. In particular, the Sennheiser microphone exhibits frequency responses more similar to the dummy ear, while the Knowles microphone seems to have slight variations in comparison with the dummy ear. Figure 15 shows the HRIRs in the time domain for the two microphones and for the five real subjects in comparison with the microphones placed on the dummy head, considering the sound source in front of the listener, i.e., $\vartheta =0^{\circ }$ and $\varphi =0^{\circ }$. Table 2 reports the LSD values comparing the HRTFs of the real subject with the HRTF measured with the mannequin at position P1. These results objectively confirm the observations already derived from the HRTFs analysis. In more detail, the worst performance is achieved by the first subject, shown in Figure 10, with the Knowles microphone. Differently, the lowest LSD value of 1.1 dB is reached by the fourth subject with the Sennheiser microphone and with the source positioned at $\vartheta =45^{\circ }$ and $\varphi =0^{\circ }$, reported in Figure 13d. Moreover, for every case, the Sennheiser microphone shows the lowest LSD value in comparison with the Knowles one and, thus, better performance.

## 5. Conclusions

In this paper, a comparative analysis of HRTFs measurement procedures is presented. In more detail, the HRTFs measured with two different in-ear microphones (i.e., Knowles and Sennheiser) are analyzed and compared with the HRTFs measured with a standard binaural mannequin. As first step, the influence of the microphone position on the frequency responses is investigated using the mannequin’s ear. Then, individual HRTFs of five real subjects are measured and compared among them. The experimental results have proven that the HRTFs are similar at low frequencies when different types of microphones are involved. In addition, the position of the microphone influences the HRTFs above 4 kHz. The experiments have shown that it is difficult to define the best position for the in-ear microphone. In fact, the analysis of the LSD values has reported that the best location of the microphone varies with the loudspeaker position and is not always the same. However, the Sennheiser microphone enables the obtaining of frequency responses more similar to the ones measured by the dummy head. Finally, the individual HRTFs measured on real subjects have shown how the frequency responses change with different ears. Furthermore, in this case, the Sennheiser microphone has produced HRTFs more similar to the mannequin and more similar among the five subjects. However, the worst performance of Knowles are compensated by the price that is one order of magnitude lower than Sennheiser microphone. Future works will investigate the effectiveness of the different HRTF measurements through subjective tests, evaluating the immersive perception. The subjective tests will examine the influence of the microphone position on the listening experience and will subjectively investigate the difference between personalized and standardized HRTFs.

## Figures and Tables

**Table 1:** LSD values (in dB) obtained in experiment 1. For each source position and microphone position, the lowest value of the LSD is bold.

Source Pos.	Mic. Pos.	Know.	Senn.
$\vartheta =0^{\circ }$, $\varphi =0^{\circ }$	P1	1.9	**1.4**
	P2	1.4	**1.1**
	P3	2.0	**1.4**
	P4	**1.5**	1.7
$\vartheta =45^{\circ }$, $\varphi =0^{\circ }$	P1	1.5	**1.1**
	P2	**1.6**	2.3
	P3	**1.3**	1.4
	P4	3.4	**1.5**
$\vartheta =0^{\circ }$, $\varphi =15^{\circ }$	P1	1.9	**1.3**
	P2	1.5	**0.9**
	P3	1.5	**1.3**
	P4	**1.6**	1.7
$\vartheta =45^{\circ }$, $\varphi =15^{\circ }$	P1	1.9	**1.7**
	P2	2.6	**1.9**
	P3	2.1	**1.3**
	P4	3.2	**1.4**

**Table 2:** LSD values (in dB) obtained in experiment 2. For each source position and subject, the lowest value of the LSD is in bold.

Source Position	Subject	Knowles	Sennheiser
$\vartheta =0^{\circ }$, $\varphi =0^{\circ }$	Subj1	2.3	**1.9**
	Subj2	1.8	**1.3**
	Subj3	2.0	**1.2**
	Subj4	2.1	**1.3**
	Subj5	1.8	**1.5**
$\vartheta =45^{\circ }$, $\varphi =0^{\circ }$	Subj1	2.7	**1.3**
	Subj2	2.0	**1.3**
	Subj3	2.2	**1.3**
	Subj4	2.1	**1.1**
	Subj4	1.6	**1.5**

## Data Availability

Not applicable.
